# Effects of Dietary Inclusion of Asiaticoside on Growth Performance, Lipid Metabolism, and Gut Microbiota in Yellow-Feathered Chickens

**DOI:** 10.3390/ani13162653

**Published:** 2023-08-17

**Authors:** Qinghua Fu, Peng Wang, Yurou Zhang, Tian Wu, Jieping Huang, Ziyi Song

**Affiliations:** Guangxi Key Laboratory of Animal Breeding, Disease Control and Prevention, College of Animal Science and Technology, Guangxi University, Nanning 530004, China; fqh8630@126.com (Q.F.); wangpeng131477@126.com (P.W.); zhangyurou0112@126.com (Y.Z.); wutian980518@126.com (T.W.); huangjieping@gxu.edu.cn (J.H.)

**Keywords:** Asiaticoside, chicken, abdominal fat, lipid metabolism, PI3K/AKT, cecal microbiota

## Abstract

**Simple Summary:**

Excessive abdominal fat deposition is a pressing problem for the poultry industry as it increases the cost of meat production. Asiaticoside (Asi) is a herbal extract, and it was found that the dietary inclusion of Asi can significantly inhibit abdominal fat deposition in chickens even at low doses. Thus, Asi presents a new promising feed additive for reducing fat deposition in the poultry industry.

**Abstract:**

Excessive abdominal fat deposition in chickens is a major concern in the poultry industry. Nutritional interventions are a potential solution, but current options are limited. Asiaticoside (Asi), a herbal extract, has shown positive effects in animals, but its impact on poultry lipid metabolism is still unknown. In this study, the effects of dietary Asi on yellow-feathered chicken lipid metabolism and its potential mechanisms were investigated. A total of 120 chickens were randomly divided into three groups, with five replicates per group and 8 chickens per replicate. The chickens were fed a basal diet supplemented with 0, 0.01, or 0.05% Asi for 6 wk. The results showed that Asi down-regulated lipogenic gene expression and up-regulated lipid-breakdown-related genes in both the liver and fat tissues of the chickens, which resulted in a half reduction in abdominal fat while not affecting meat yield. Mechanistically, the hepatic and adipose PI3K/AKT pathway may be involved in Asi-induced fat loss in chickens as revealed by computer-aided reverse drug target prediction and gene expression analysis. Moreover, Asi ingestion also significantly modified the cecal microbiota of the chickens, resulting in a reduced *Firmicutes* to *Bacteroidetes* ratio and decreased abundance of bacteria positively correlated with abdominal fat deposition such as *Ruminococcus*, while increasing the abundance of bacteria inversely correlated with abdominal fat deposition such as *Lactobacillus*, *Bacteroides*, and *Blautia*. Collectively, these data suggest that Asi could ameliorate the abdominal fat deposition in yellow-feathered chickens, probably through modulating the PI3K/AKT pathway and gut microbiota function.

## 1. Introduction

Poultry products are rich in highly digestible protein (with low levels of collagen), B vitamins, and minerals, which are essential components of a balanced diet for human beings [1]. However, selective breeding for growth has led to excessive fat deposition in chickens, comprising up to 20% of their body weight [2]. This over-deposition of fat reduces feed efficiency, increases meat production costs, and adversely affects consumer health [3]. Consequently, managing excessive fat deposition has become a critical challenge in chicken farming.

The regulation of abdominal fat accumulation in chickens is determined by the balance between triglycerides synthesis/uptake and breakdown [4]. The liver, which is responsible for synthesizing over 90% of the de novo fatty acids in poultry, is a vital target in managing abdominal fat accumulation [5]. Furthermore, gut microflora has a significant impact on host lipid metabolism, and its composition varies depending on diet, medications, and other environmental factors [6,7]. Therefore, manipulating the gut microbiota is another promising strategy for regulating fat accumulation in chickens.

In recent years, the use of antibiotics as growth promoters in animal feeds has been banned in many countries [8,9]. Consequently, Chinese herbs and their extracts have emerged as a potential alternative [10]. Asiaticoside (Asi), the main glycosidic compound extracted from *Centella asiatica* (L.) *Urban*, is well known for its cosmetic effects, anti-inflammatory properties, and promoting wound healing [11,12,13]. Moreover, Asi has been shown to have an anti-diabetic effect in a high-fructose-fat-fed rat model [14]. Furthermore, Asi has been reported to reduce autophagy and improve memory in dementia model rats by blocking the mechanistic target of the rapamycin (mTOR) pathway [15].

Although several studies have been carried out, it is not clear whether Asi plays a role in lipid metabolism. Hence, the aim of this study was to investigate the effects of dietary Asi at different doses on abdominal fat deposition in chickens and its underlying mechanisms. Our results will provide new insights to address the challenge of lipid over-accumulation in poultry production. 

## 2. Materials and Methods

### 2.1. Animals, Diets, and Experimental Design

The Animal Ethics Committee at Guangxi University approved our animal studies (GXU-2022-022). One hundred and twenty 21-day-old yellow-feathered chickens were randomly divided into three groups, with five replicates per group and eight chickens per replicate. The chickens were fed either a basal diet or a basal diet supplemented with different concentrations of Asi powder (0.01% or 0.05%) for 42 days. The Asi used in the study had a purity of ≥70% and was purchased from Chengtai Biotechnology Co. (Nanning, China). The feeds used for the experiments were grower feed obtained from Nanning Weimin Feed Co. (Nanning, China) as previously described [16], and their composition is shown in Table 1. The chickens were housed in an environmentally controlled room at the Animal Experiment Center of Guangxi University and provided ad libitum access to food and water throughout the experimental period.

### 2.2. Measurement of Growth Performance

Body weight and feed intake were measured every two weeks to evaluate the effect of dietary Asi on growth performance. The average daily feed intake (ADFI) was calculated based on the feed consumed.

### 2.3. Slaughter Indicators and Organ Index

At the end of the experiment, two chickens with body weights close to the group mean were selected from each of the five replicates in each group, fasted for 12 h, and then slaughtered. The following parameters were measured: slaughter rate, eviscerated rate, chest muscle rate, thigh muscle rate, subcutaneous fat thickness, intermuscular fat width, and abdominal fat weight. The slaughter rate was calculated as the weight of the chicken after bleeding and dehairing divided by the fasting body weight multiplied by 100%. The eviscerated rate was calculated as the weight of the chicken left after removing the trachea, esophagus, shivering sac, intestines, spleen, pancreas, heart, liver, glandular stomach, muscular stomach, abdominal fat, and reproductive organs divided by the fasting body weight multiplied by 100%. Intermuscular fat width (IFW) referred to the width of the fat bands between the axilla and sternum along the pectoralis major edge, and subcutaneous fat thickness (SFT) was determined by making a transverse cut at the caudal union and then a longitudinal cut from the dorsal midline. The fat thickness was measured at the intersection with a vernier caliper. The abdominal fat rate (AFR) was calculated using the following formula: abdominal fat rate (%) = abdominal fat weight/(abdominal fat weight + eviscerated weight) × 100.

### 2.4. Sample Collection

Blood samples were collected from the wing veins at the time of slaughter, and the serum was obtained by centrifuge at 3000× *g* for 10 min at 4 °C. Chickens with body weights close to the group mean (10 per group) were selected and euthanized by cervical dislocation. The collected samples were immediately frozen using liquid nitrogen and stored at −80 °C for subsequent analysis.

### 2.5. Determination of Serum Biochemical Indicators and Liver Parameters

The assay kits (A110-1-1, A111-1-1, A112-1-1, and A113-1-1, Nanjing Jiancheng Bioengineering Institute, Nanjing, China) and their instructions were used to measure triglycerides (TG), total cholesterol (TC), high-density lipoprotein cholesterol (HDL-C), and low-density lipoprotein cholesterol (LDL-C).

### 2.6. Hematoxylin and Eosin (h&e) Staining

Liver and adipose tissues fixed overnight in 4% fixative solution were sent to Wuhan Servicebio Biotechnology Co., Ltd. (Wuhan, China) for paraffin embedding, sectioning, and staining. Images were taken at different magnifications using a light microscope (Biological microscope ML31; MSHOT, Guangzhou, China), and cell areas were counted using Image J software (v1.53t).

### 2.7. RNA Extraction and Quantitative Real-Time PCR

The total RNA of liver and adipose tissues was extracted using a previously described method [17]. The All-in-One First-Strand cDNA Synthesis SuperMix (AE341-02, Transgen, Beijing, China) was used to obtain cDNA from 1 μg of total RNA. Quantitative real-time polymerase chain reaction (RT-qPCR) was performed using the 2× RealStar Green Fast Mixture (A301-01, GenStar, Beijing, China), and relative gene expression levels were normalized to β-actin. The primer sequences used in the RT-qPCR are provided in Table 2. The 2^−ΔΔCT^ method was used to analyze the relative gene expression data.

### 2.8. Western Blotting

Western blot analysis was performed as previously described [18]. Briefly, frozen liver and adipose tissues from 3 chickens slaughtered per group were lysed using RIPA lysis buffer (IN-WB001, Invent, Plymouth, MA, USA) with 1 mM PMSF (P0100, Solarbio Technology Co., Beijing, China). The lysates were then diluted with Sample Loading Buffer (P0015L, Beyotime, Shanghai, China), boiled, and loaded onto an 8% SDS–PAGE. After electrophoresis, the proteins were transferred to a PVDF membrane and the membranes were routinely washed and blocked with 5% non-fat dry milk in TBST. Then, the following primary antibodies were used: anti-mTOR (T55306F, 1:1000 dilution), anti-phospho-AKT (Ser473) (T40067F, 1:2000 dilution), anti-AKT (T55561F, 1:2000 dilution), and anti-β-Tubulin (M20005F, 1:5000 dilution), all purchased from Abmart Shanghai Co., Ltd. (Shanghai, China). After applying the secondary antibodies, the membranes were developed using chemiluminescence (Abbkine, Waltham, MA, USA) and the signals were detected using a Bio-Rad Imaging System (Bio-Rad, Hercules, CA, USA).

### 2.9. Reverse Screening and KEGG Analysis

The 3D structure of Asi was downloaded from ChemSpider (http://www.chemspider.com/, accessed on 17 June 2022). The Ligand Profiler program in Discovery Studio 2020 (DS, Accelrys, San Diego, CA, USA) was used to predict the target of Asi in PharmaDB, which is the largest database of receptor–ligand complex pharmacophores for inverse target discovery [19]. The Protein Data Bank (PDB) IDs of predicted target proteins (Fit value > 0.6) were annotated with Biomart (v2.50.0) to obtain the corresponding gene IDs. The gene IDs were then analyzed by Cluster Profiler (v3.0.4) for the Kyoto Encyclopedia of Genes and Genomes (KEGG), and the results were visualized using ggplot2 (v3.3.6).

### 2.10. Cecal Microbiome Analysis by 16S rDNA Sequencing

The cecal contents were collected and sent to Shanghai Biozeron Biotechnology Co., Ltd. (Shanghai, China) for 16S rDNA sequencing. Total genomic DNA was extracted using the CTAB/SDS method. The V3 and V4 regions of the 16S rDNA gene were amplified using primers 341F (5′-CCTAYGGGRBGCASCAG-3′) and 806R (5′-GGACTACNNGGGTATCTAAT-3′). Sequencing was conducted on the Illumina MiSeq PE300 Platform/NovaSeq PE250 Platform (San Diego, CA, USA).

16S rDNA sequencing analysis was performed as previously described [17]. Briefly, the obtained sequence files were analyzed using QIIME2 (v2021.2.0). After normalization to obtain 40,000 signature sequences per sample, the DADA2 module of QIIME2 was used to perform noise reduction and create an amplicon sequence variation (ASV) table. Untrained sequence files were downloaded from GreenGenes (https://greengenes.lbl.gov, accessed on 20 July 2022), and a naive Bayesian classifier was used to generate the designated region based on sequencing primers. The 16S rDNA genes were then annotated using trained feature sequences.

The α-diversity of microbiome was described by using indices of Shannon, Simpson, Chao1, and ACE with Vegan (v2.5-7, R package), and β-diversity of microbiome was evaluated with Phyloseq (v1.3.20, R package). The taxonomic abundance of specific genera was summarized at the phylum and genus levels using Phyloseq (v1.13.0, R package). Linear discriminant analysis (LDA) and effect size (LEfSe) methods and non-parametric Kruskal–Wallis rank sum tests were employed to identify different groups in multiple samples. Pearson correlation analysis was used to show the correlation between bacterial abundance and abdominal fat rate, serum TG levels, liver TG levels, and lipid-metabolism-related gene expression levels. All results were visualized using the R package ggplot2 (v3.3.6).

### 2.11. Statistical Analysis

The results are expressed as means ± SEM. Significance was estimated via a one-way ANOVA of SPSS 26.0. Tukey post hoc analysis was conducted to test the level of significance. Polynomial orthogonal contrasts were used to determine the linear and quadratic responses of measured parameters to dietary Asi concentrations. A probability of <0.05 was considered to be statistically significant. The histogram was prepared using GraphPad Prism 8.0.

## 3. Results

### 3.1. Effect of Dietary Asi on Growth Performance

The impact of Asi on chicken growth performance was evaluated and shown in Table 3. Asi ingestion did not affect feed intake (*p >* 0.05) but significantly attenuated bodyweight growth during the trial, particularly on days 28 to 42 for both treatments (*p <* 0.05) and showed a significant linear and quadratic relationship (*p* < 0.05). Importantly, after slaughter, we found that Asi addition had no effect on the slaughtering and eviscerated rates (*p* > 0.05). Additionally, the absolute weights of the chest and thigh muscles were not affected by Asi (*p* > 0.05), but the relative ratio was increased. Collectively, these data suggest that dietary Asi decreases the body weight but not the meat yield of chickens.

### 3.2. Effect of Dietary Asi on Abdominal Fat Deposition

**Figure 1:**
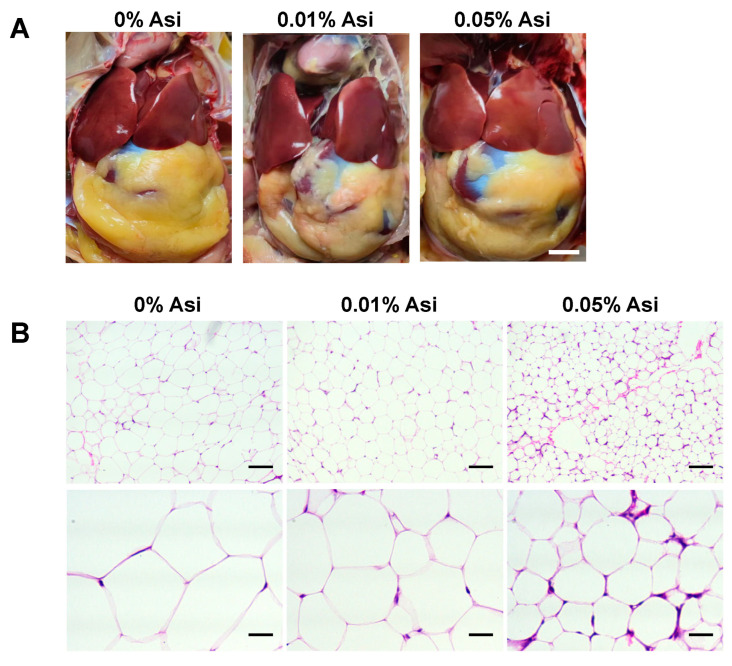
The effects of dietary Asi on abdominal fat deposition of chickens. (**A**) Representative pictures of chickens. Scale bar, 1 cm. (**B**) Representative images of abdominal adipose tissue with H&E staining viewed under a microscope (200× and 800×). Scar bars 100 μm and 25 μm.

Dissection revealed a visually reduced abdominal fat pad in chickens with Asi treatment (Figure 1A). Furthermore, Asi decreased abdominal fat deposition in a dose-dependent manner (*p* < 0.001, Table 4). Along with the reduction in abdominal fat, the thickness of subcutaneous fat was also significantly reduced (*p* < 0.05, Table 4); however, the width of intermuscular fat was not affected (*p* > 0.05, Table 4). Consistent with our previous observations, H&E staining of fat tissues revealed that dietary Asi clearly reduced the areas of the abdominal fat cells (*p* < 0.05, Figure 1B, Table 4). Overall, dietary Asi significantly reduced abdominal fat deposition in chickens.

### 3.3. Effect of Dietary Asi on Hepatic Lipid Deposition and Serum Lipid Metabolism

**Figure 2:**
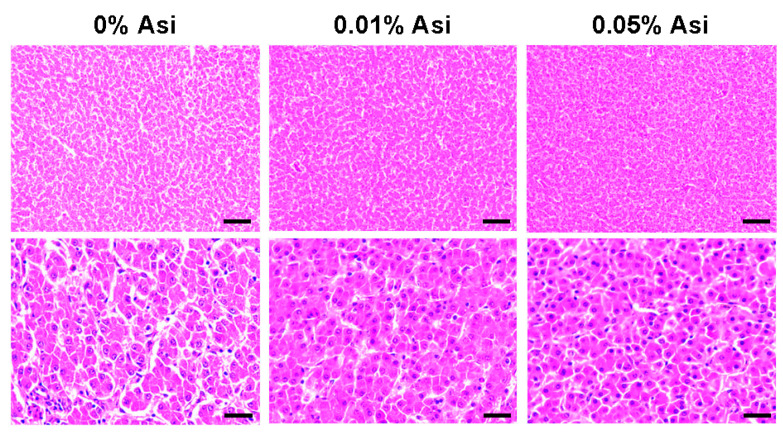
Representative images of liver tissue with H&E staining viewed under a microscope (200× and 800×). Scar bars 100 μm and 25 μm.

Given the crucial role the liver plays in lipid metabolism, the effects of Asi on hepatic lipid metabolism were investigated. As shown in Table 5, dietary Asi induced a small but significant decrease in liver weight (*p* < 0.01) in chickens, which may be due to the significant reduction in the TG content, although no apparent morphological changes were observed (Figure 2). To further investigate the impact of Asi on the lipid metabolism of chickens, lipid indices in the serum were measured (Table 5). The results showed that 0.05% Asi reduced serum TG levels by 32% (*p* < 0.01) and LDL-C levels by 52% (*p* < 0.01) with a significant linear relationship (*p* < 0.01). Interestingly, Asi had no effect on TC (*p* > 0.05) and HDL-C (*p* > 0.05) levels. Together, these data suggest that dietary Asi can effectively improve lipid metabolism in both the liver and serum of chickens.

### 3.4. Effect of Dietary Asi on PI3K/AKT Pathway

**Figure 3:**
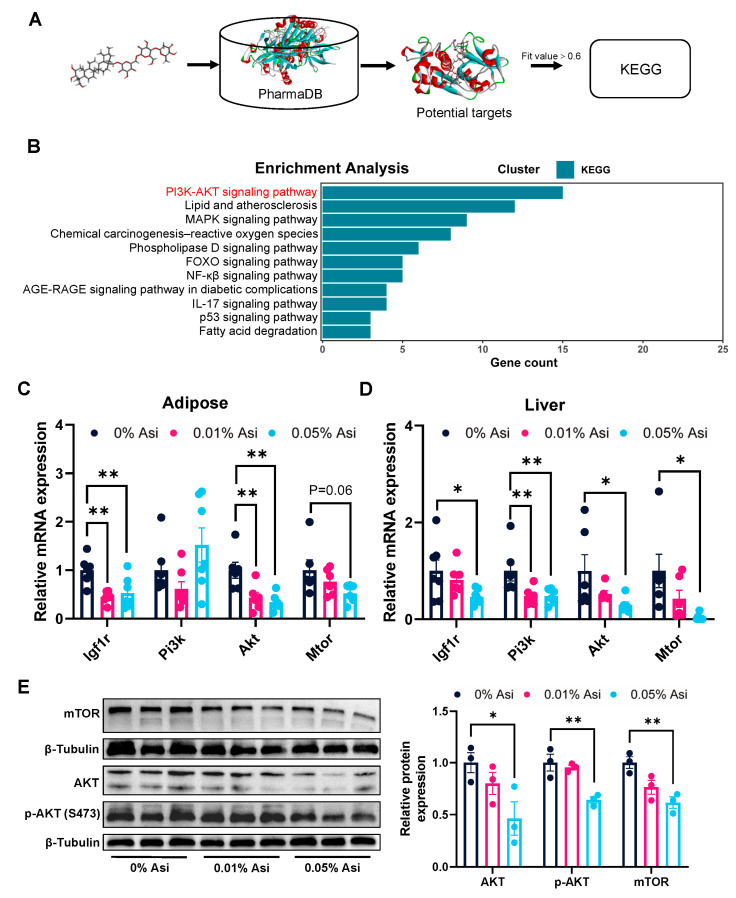
Reverse targets prediction, KEGG pathway analysis, and validation of the Asi targets. (**A**) Flow chart of reverse target finding. (**B**) KEGG analysis of potential targets. (**C**) Relative mRNA expression in adipose (*n* = 6–8). (**D**) Relative mRNA expression in liver (*n* = 6–8). (**E**) Protein of PI3K/AKT signaling pathway genes were detected by Western blotting (*n* = 3). All the results are shown as the means ± SEM; * *p* < 0.05, ** *p* < 0.01.

To investigate the molecular mechanisms underlying the beneficial effects of Asi on lipid metabolism, computer-aided reverse drug target prediction was applied to identify the potential protein targets of Asi, as described in Figure 3A. A total of 99 proteins with a Fit value > 0.6 were predicted as potential targets of Asi. To further identify pathways that might be involved in the lipid-lowering effects of Asi, the KEGG pathway analysis was conducted on the 99 protein candidates (Figure 3B). Interestingly, the most enriched pathway was the PI3K/AKT signaling pathway, a well-known pathway that regulates lipid metabolism through its downstream targets of the mTOR complex and FOXO1 transcription factor [20].

Then, to investigate the regulatory role of Asi on the PI3K/AKT pathway, qRT-PCR analysis was performed to measure the expression of genes associated with this pathway. The results showed that Asi treatment led to the down-regulation of *Igf1r* and *Akt* in both the abdominal fat and liver tissues of chickens (*p* < 0.05, Figure 3C, D). Furthermore, the protein levels of hepatic AKT were decreased by 0.05% Asi treatment (Figure 3E). In agreement with the total AKT levels, the levels of phospho-AKT (Ser473) were also down-regulated (Figure 3E). In addition, consistent with the *Mtor* mRNA levels, a reduction in the total mTOR protein levels was observed (Figure 3E). These findings tentatively suggest that the PI3K/AKT pathway could be involved in the mechanism mediating Asi-induced fat reduction in chickens.

### 3.5. Effect of Dietary Asi on the Expression of Genes Involving in the Lipogenesis and Lipid Breakdown

**Figure 4:**
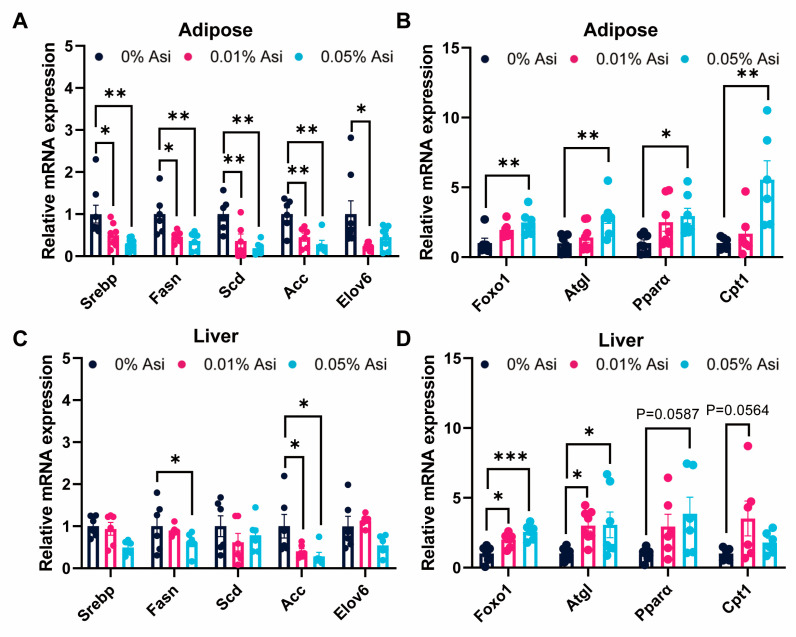
The effects of dietary Asi on lipid-metabolism-related gene expression in abdominal fat and liver of the chickens. (**A**) Relative mRNA related to lipid synthesis expression in adipose. (**B**) Relative mRNA related to lipolysis expression in adipose. (**C**) Relative mRNA related to lipid synthesis expression in liver. (**D**) Relative mRNA related to lipolysis expression in liver. All the results are shown as the means ± SEM; *n* = 6–8; * *p* < 0.05, ** *p* < 0.01, *** *p* < 0.001.

To further investigate the molecular mechanisms underlying the fat-lowering effects of Asi in chickens, the downstream targets of the PI3K/AKT pathway involved in lipid metabolism were examined (Figure 4). Consistent with our previous findings, dietary Asi led to a significant reduction in the mRNA levels of genes responsible for lipid biosynthesis, including the lipogenic transcription factor *Srebp* and its target genes *Fasn*, *Scd*, *Acc*, and *Elov6* in abdominal fat tissue following Asi ingestion (*p* < 0.05). And *Fasn* and *Acc* showed the same trend in liver tissue (*p* < 0.05). Furthermore, the addition of Asi increased the expression of genes involved in lipolysis and lipid β-oxidation, such as *Foxo1*, *Atgl*, *Pparα*, and *Cpt1*, in both abdominal fat (*p* < 0.05) and liver (*p* < 0.05). Taken together, these findings suggest that the inhibition of lipid biosynthesis and the promotion of lipolysis and lipid β-oxidation in the liver and abdominal fat tissues are responsible for Asi-induced fat reduction in chickens.

### 3.6. Effect of Dietary Asi on Cecal Microbiota

**Figure 5:**
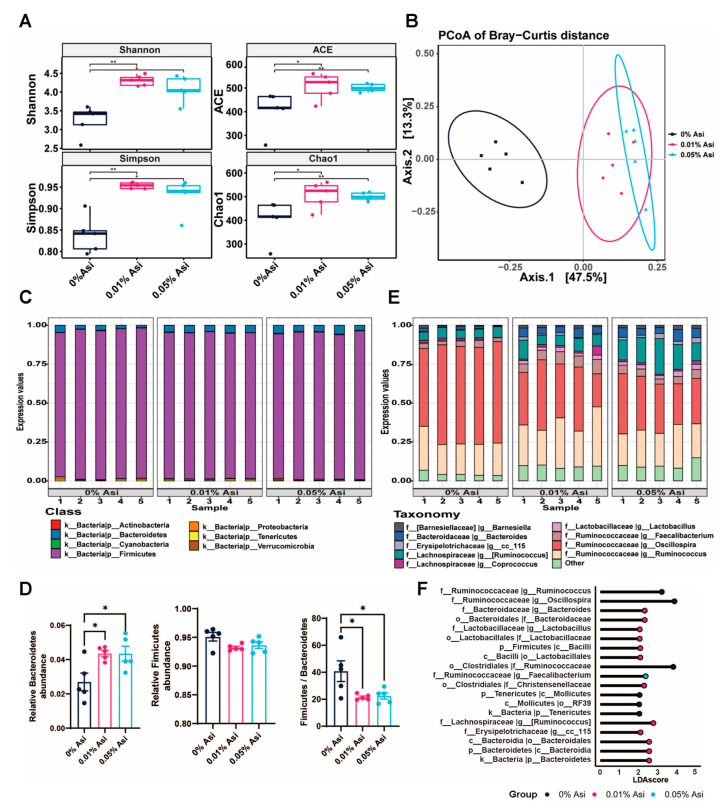
The effects of dietary Asi on diversity and composition of the cecum microbiota in chickens. (**A**) Alpha diversity. (**B**) PCoA analysis results. (**C**) Species distribution at the phylum level. (**D**) The ratio of *Fimicutes* and *Bacteroides*. (**E**) Species distribution at the genus level. (**F**) LDA discrimination. All the results are shown as the means ± SEM; *n* = 5; * *p* < 0.05, ** *p* < 0.01.

Intestinal flora has been shown to be closely related to fat metabolism [21,22,23]. To comprehensively understand the mechanisms underlying the lipid-lowering effects of Asi, the impact of dietary Asi on the composition of the gut microbiota was evaluated using 16s rDNA sequencing. The α-diversity of the intestinal flora at the operational taxonomical units (OTUs) level was investigated by indices of Simpson and Shannon as well as the abundance-based coverage estimator (ACE) and Chao1, which indicate the diversity and richness of the intestinal flora within the group, respectively. Compared to the control group, Asi significantly increased the α-diversity of the cecal microorganisms (Figure 5A). Subsequently, UniFrac PCoA analysis for β-diversity of the intestinal flora revealed differences in microbial composition between the control and Asi treatment groups (Figure 5B).

To obtain detailed information on microbial composition, the OTUs corresponding to species were annotated and visualized by levels of phylum and genus (Figure 5C–E). In all three groups, *Firmicutes* and *Bacteroidetes* were the dominant microbial communities at the phylum level, but their ratio was significantly reduced after Asi treatment. At the genus level, *Oscillospora*, *Ruminococcus*, and *Bacteroidetes* were the common predominant bacteria, and their relative ratios were shifted by dietary Asi. Finally, an LFfSe analysis was conducted to further identify differences in the abundance of microbes between the control and treatment groups (Figure 5F). *Oscillospira*, *Ruminococcaceae*, etc., were the dominant genera in the control group, whereas Bacteroides dominated in the treatment groups. In summary, these data suggest that dietary Asi remodels the diversity and dominant bacteria composition of the cecal microbiota in chickens.

### 3.7. Correlation of Gut Microbiota with Metabolic Phenotypes

**Figure 6:**
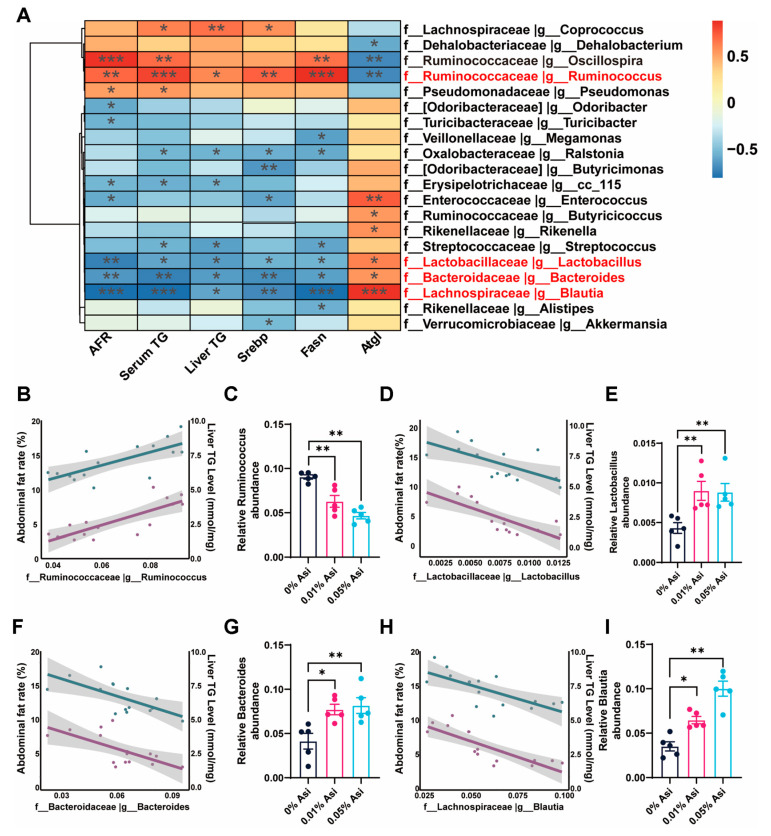
Correlation analysis. (**A**) Pearson correlation analysis. (**B**) The correlation between *Ruminococcus* and abdominal fat rate and liver TG. (**C**) Relative *Ruminococcus* abundance of cecal. (**D**) The correlation between *Lactobacillus* and abdominal fat rate and liver TG. (**E**) Relative *Lactobacillus* abundance of cecal. (**F**) The correlation between *Bacteroides* and abdominal fat rate and liver TG. (**G**) Relative *Bacteroides* abundance of cecal. (**H**) The correlation between *Blautia* and abdominal fat rate and liver TG. (**I**) Relative *Blautia* abundance of cecal. All the results are shown as the means ± SEM; * *p* < 0.05, *n* = 5; ** *p* < 0.01; *** *p* < 0.001.

In order to investigate the functional interactions between gut microbiota and host lipid metabolism, a Pearson correlation analysis between microbiota abundance and metabolic phenotypes, including abdominal fat rate (AFR), serum triglyceride (TG) levels, liver TG levels, and lipid-metabolism-related gene expression levels, was performed. The results showed that there was a significant correlation between gut microbiota abundance and metabolic parameters (Figure 6A). For instance, *Ruminococcus*, which has been reported to be positively correlated with fat accumulation [24], showed a positive correlation with AFR and liver TG levels, but its abundance was actually reduced by Asi addition (*p* < 0.01, Figure 6B,C). Conversely, *Lactobacillus*, *Bacteroides*, and *Blautia*, which have been reported to be negatively correlated with fat deposition [25,26,27], showed a negative correlation with AFR and liver TG levels, but their abundance was significantly elevated by Asi addition (*p* < 0.05 or 0.01, Figure 6D–I). These results suggest that the Asi-induced gut microbiota alteration may contribute to the improvement of lipid metabolism in chickens.

## 4. Discussion

Excessive fat deposition in chickens is a major issue in chicken production, which can reduce feed conversion and edible carcass yield [28]. Traditional Chinese medicine is emerging as a potential source of new chemicals to combat this problem [29]. Asiaticoside (Asi), derived from the Chinese medicinal herb *Centella asiatica* (L.) *Urban*, has multiple beneficial effects including immunomodulation, anti-inflammation, anti-oxidation, anti-diabetes, and promoting wound healing [13,30,31]. However, the effects of Asi on chicken lipid metabolism are unclear. To address this gap, we investigated the effects of the dietary addition of Asi on chicken fat deposition and its potential mechanisms.

A dietary addition of Asi for six weeks did not affect the meat yield of chickens (*p* > 0.05, Table 3) but specifically reduced abdominal and liver fat deposition in a dose-dependent manner (Table 4 and Table 5, Figure 1), indicating Asi’s potential as a lipid-lowering compound in chickens. Consistent with our findings, a recent study showed that dietary madecassoside, a derivative of Asi, inhibited fat accumulation in a high-fat-diet-fed mouse model [32]. Therefore, Asi may play an inhibitory role in fat deposition across species. Further investigations could be conducted in layer chickens, pigs, or even in clinical trials to expand the scope and significance of Asi in fat metabolism.

To explore the mechanism underlying Asi’s effects on fat reduction, a computer-aided target recognition technology was used to identify potential protein targets of Asi. KEGG pathway analysis revealed that these targets were mainly enriched in the PI3K/AKT signaling pathway, which plays a critical role in regulating lipid metabolism in both liver and adipose tissue [33]. Interestingly, Asi has been reported to regulate the PI3K/AKT pathway in neuroblast-like cell lines and cardiomyocytes [34], indicating that our reverse screening strategy was reliable. To support this prediction, two pieces of evidence were presented. First, a significant reduction in the mRNA levels of major genes related with the PI3K/AKT signaling pathway was observed in the presence of 0.05% Asi in both liver and abdominal fat (*p* < 0.05, Figure 3C–E). Consistently, the total protein levels of AKT and mTOR in the liver were also down-regulated after Asi treatment (*p* < 0.05, Figure 3E). Nevertheless, to strengthen this evidence, the protein levels of PI3K, phospho-AKT at other sites besides S473, and the mTOR complex components should be detected in future studies. Second, the expression of downstream targets of the PI3K/AKT pathway was also regulated by dietary Asi in both liver and abdominal fat. Notably, previous studies have shown that the activation of the PI3K/AKT pathway promotes lipid synthesis and inhibits lipid breakdown through the modulation of mTOR complex activities [20]. Consistent with this, genes related to lipid biosynthesis were down-regulated (*p* < 0.05, Figure 4A,C), while genes involved in lipid breakdown were up-regulated in both liver and abdominal fat (*p* < 0.05, Figure 4B,D). Overall, while the possibility of other pathways contributing to the observed effects cannot be excluded, the data suggest that the PI3K/AKT pathway in the liver and abdominal fat may be involved in Asi-induced fat loss in chickens.

To gain a comprehensive understanding of the mechanisms underlying Asi-induced fat reduction in chickens, the indirect mechanism involving the role of gut microbiota was also investigated. This is based on the fact that gut microbiota can influence a range of host factors, from gut structure and function to nutrient status, and that Chinese herb extracts have been shown to modulate the metabolism of humans and animals through the gut microbiota [35,36]. Consistent with these findings, dietary Asi improved the diversity and composition of the cecum microbiota (Figure 5). Specifically, the addition of Asi decreased the abundance of *Ruminococcus* (*p* < 0.05, Figure 6B,C) while increasing the abundance of *Lactobacillus*, *Bacteroides*, and *Blautia* (*p* < 0.05, Figure 6D–I), which have been reported to be positively or negatively correlated with fat deposition [24,25,26,27]. Although the detailed mechanisms remain unclear, short-chain fatty acids (SCFAs) such as acetate, propionate, and butyrate produced by gut microbiota are known to have beneficial effects on the host [37]. Therefore, it is possible that SCFAs mediate the role of the modified gut microbiota in promoting lipid amelioration by Asi in chickens. Additionally, it is plausible that the inhibition of the PI3K/AKT pathway by Asi is mediated by SCFAs, particularly by butyrate, since sodium butyrate has been shown to inhibit the PI3K/AKT/mTOR signaling pathway in enteroendocrine cells [38]. However, further research is needed to confirm these speculations.

Of note, although 0.05% of Asi may be the optimal dose among the three treatments, more studies are required to figure out the best dose of Asi for poultry production. Moreover, yellow-feathered chickens are a slow-growing breed, with fat deposition mainly occurring in the later stages of growth. In this study, high-energy diets were used to accelerate the fat deposition process of the chickens and shorten the trial period. While the regulation of lipid metabolism is conserved among breeds, it is still necessary to test the effects of Asi on other chicken breeds before the widespread application of Asi as a feed additive in chicken production.

## 5. Conclusions

In summary, our study reveals that the dietary inclusion of 0.05% Asi can effectively reduce the deposition of abdominal fat in yellow-feathered chickens, which is the first study of Asi in chicken research. The underlying mechanisms probably involve inactivating the PI3K/AKT signaling pathway and optimizing the cecal microbiota. After verifying the effects of Asi on other chicken breeds, the findings here could translate into substantial economic benefits for industrial chicken production, and also shed light on the potential of herbal extracts in regulating complex traits in chickens.

## Figures and Tables

**Table 1:** Composition of basal diets.

Ingredients	Percentage (%)
Corn	67.546
Soybean protein	1.804
Soybean meal	9.500
Wheat bran	4.500
Rice bran	4.500
CaCO_3_	2.255
CaHPO_4_	1.804
Pig tallow	5.402
NaCl	0.363
DL-Methionine	0.363
Premix ^1^	0.005
Cholesterol	1.961
Nutrient composition ^2^	
ME (MJ/kg)	13.052
Crude protein	12.976
Lys	0.600
Met	0.590

^1^ Premix (per kg of diet): Vit A, 6000 IU; Vit D3, 200 IU; Vit E, 10 IU; Vit K, 0.50 mg; Vit B12, 0.007 μg; pantothenic acid, 2.99 mg; riboflavin, 1.63 mg; Cu, 1.25 mg; Mn, 24.06 mg; Zn, 12.7 mg; Se, 0.06 mg; iodide, 0.35 mg. ^2^ Values were calculated from data provided by Feed Database in China (2020).

**Table 2:** Primers used for a quantitative polymerase chain reaction.

Gene Name	Gene ID	Primer (5′-3′)	Product Size, bp
*β-actin*	NM_205518.1	F: TGCGTGACATCAAGGAGAAG	300
		R: TGCCAGGGTACATTGTGGTA	
*Igf1r*	NM_205032	F: CTGTGTCCGACAAATGGGGA	169
		R: TGACGGTCAGTTTCGGGAAG	
*Pi3k*	XM_049803139	F: GCATCAGTGGCTCAAGGACA	80
		R: AGCCAGCACAAGAACGTGTA	
*Akt*	XM_015274151	F: GAAGTGCTGGAGGACAACGA	103
		R: CCTGGTTGTAGAAGGGCAGG	
*Mtor*	XM_417614	F: GGTGATGACCTTGCCAAACT	220
		R: CTCTTGTCATCGCAACCTCA	
*Srebp*	XM_015294109	F: GCCCTCTGTGCCTTTGTCTTC	130
		R: ACTCAGCCATGATGCTTCTTC	
*Fasn*	NM_205155.4	F: TGAAGGACCTTATCGCATTGC	96
		R: GCATGGGAAGCATTTTGTTGT	
*Scd*	NM_204890	F: GTTTCCACAACTACCACCATACATT	175
		R: CCATCTCCAGTCCGCATTTT	
*Acc*	J03541	F: GCTTCCCATTTGCCGTCCTA	185
		R: GCCATTCTCACCACCTGATTACTG	
*Elov6*	NM_001031539	F: GGTGGTCGGCACCTAATGAA	169
		R: TCTGGTCACACACTGACTGC	
*Foxo1*	NM_204328	F: AAGAGCGTGCCCTACTTCAA	125
		R: TTCCCTGTTCCCTCATTCTG	
*Atgl*	NM_001113291	F: TCCTAGGGGCCTACCACATC	195
		R: CCAGGAACCTCTTTCGTGCT	
*Pparα*	NM_001001464.1	F: TGCTGTGGAGATCGTCCTGGTC	166
		R: CTGTGACAAGTTGCCGGAGGTC	
*Cpt1*	DQ314726.1	F: GCCAAGTCGCTCGCTGATGAC	166
		R: ACGCCTCGTAGGTCAGACAGAAC	

**Table 3:** The effects of dietary Asi on growth performance of chickens.

Item	Treatment			SEM ^1^	Statistics		
	0% Asi	0.01% Asi	0.05% Asi		*P_anova_*	*P_linear_*	*P_quadratic_*
BW 0 d, g	241.52	238.06	240.03	2.98	0.720	0.929	0.430
BW 14 d, g	388.31 ^a^	373.84 ^a^	366.11 ^b^	5.11	0.028	0.018	0.155
BW 28 d, g	589.38 ^a^	539.05 ^b^	512.68 ^b^	8.67	<0.001	<0.001	0.009
BW 42 d, g	895.88 ^a^	856.15 ^b^	832.52 ^c^	4.73	<0.001	<0.001	0.001
ADFI, g/day	61.38 ^ab^	58.20 ^b^	62.83 ^a^	1.06	0.026	0.080	0.026
Slaughtering rate, %	94.85	96.95	95.99	0.73	0.146	0.631	0.059
Eviscerated rate, %	56.08	57.37	55.85	0.72	0.288	0.468	0.162
Chest muscle, g	40.63	40.16	42.08	1.18	0.715	0.459	0.737
Chest muscle, %	8.04 ^b^	9.51 ^a^	10.08 ^a^	0.43	<0.001	0.001	0.016
Thigh muscle, g	62.40	62.57	62.92	1.95	0.982	0.851	0.980
Thigh muscle, %	13.49 ^b^	13.31 ^b^	14.42 ^a^	0.23	0.005	0.002	0.230

Abbreviations: BW = body weight; ADFI = average daily food intake. ^1^ SEM = standard error of mean, *n* = 8–10. ^a–c^ Values within a row with different superscripts differ significantly at *p* < 0.05.

**Table 4:** The effects of dietary Asi on fat deposition.

Item	Treatment			SEM ^1^	Statistics		
	0% Asi	0.01% Asi	0.05% Asi		*P_anova_*	*P_linear_*	*P_quadratic_*
AFW, g	35.13 ^a^	17.60 ^b^	13.51 ^b^	2.71	<0.001	<0.001	0.001
AFR, %	6.80 ^a^	3.65 ^b^	3.06 ^b^	0.51	<0.001	<0.001	0.001
SFT, mm	6.80 ^a^	4.56 ^b^	3.75 ^b^	0.39	0.001	0.001	0.049
IFW, mm	9.40	8.62	8.19	0.94	0.404	0.249	0.492
Adipocyte area ^2^, μm^2^	3573.93 ^a^	2433.61 ^b^	1736.00 ^b^	227.7	0.004	0.002	0.040

Abbreviations: AFW = abdominal fat weight; AFR = abdominal fat rate; SFT = subcutaneous fat thickness; IFW = intermuscular fat width. ^1^ SEM = standard error of mean, *n* = 8–10. ^2^ Mean values are based on H&E staining of abdominal adipose tissue (*n* = 3). ^a,b^ Values within a row with different superscripts differ significantly at *p* < 0.05.

**Table 5:** The effects of dietary Asi on liver lipid deposition.

Item	Treatment			SEM ^1^	Statistics		
	0% Asi	0.01% Asi	0.05% Asi		*P_anova_*	*P_linear_*	*P_quadratic_*
Liver							
Liver weight/BW, %	2.26 ^a^	2.07 ^b^	1.99 ^b^	0.06	0.009	0.008	0.083
TG, mmol/mg	15.87 ^a^	14.70 ^a^	12.07 ^b^	0.68	0.001	<0.001	0.625
Serum							
TG, mmol/L	0.37 ^a^	0.28 ^b^	0.25 ^b^	0.02	<0.001	0.001	0.017
TC, mmol/L	5.02	5.30	4.90	0.26	0.561	0.525	0.390
HDL-C, mmol/L	2.20	2.27	2.45	0.39	0.439	0.207	0.899
LDL-C, mmol/L	2.24 ^a^	1.54 ^b^	1.07 ^b^	0.94	<0.001	<0.001	0.054

Abbreviations: TG = triglyceride; TC = total cholesterol; HDL-C = high-density lipoprotein cholesterol; LDL-C = low-density lipoprotein cholesterol. ^1^ SEM = standard error of mean, *n* = 8–10. ^a,b^ Values within a row with different superscripts differ significantly at *p* < 0.05.

## Data Availability

The data that support the findings of this study are available in the BioProject dataset at https://www.ncbi.nlm.nih.gov/bioproject/ (accessed on 15 September 2022), reference number: PRJNA880753.
